# Cardiac MRI identifies the possible cause of sudden cardiac arrest in more than 50% of resuscitated patients

**DOI:** 10.1186/1532-429X-15-S1-P254

**Published:** 2013-01-30

**Authors:** Umesh C Sharma, Nirmal Kharel, Yeong Shyan Lee, Brian B Ghoshhajra, Suhny Abbara

**Affiliations:** 1 Radiology, MGH, Boston, MA, USA; 2 Medical Oncology, Dana Farber Cancer Institute, Boston, MA, USA

## Background

Sudden cardiac arrest (SCA) results from malignant ventricular arrhythmias that may be due to myocardial ischemia, infarct, edema, fibrosis or infiltration. The underlying cause is often unknown. Cardiac magnetic resonance (CMR) may provide structural and functional data and identify myocardial ischemia, inflammation and fibrofatty infiltration.

This study determines the diagnostic ability of CMR in SCA of unknown cause.

## Methods

Seventy-six patients with SCA of unknown cause underwent CMR. The results were adjudicated into either non-diagnostic (no convincing cause identified) or diagnostic CMR.

## Results

Of the 76 CMR studies, 41 (52%) demonstrated potentially causative pathology for SCA. Of these 41 patients- 11 (14%) had recent ischemia or major LV wall motion defects, 7 (9%) had myocarditis, 6 (7%) had arrhythmogenic right ventricular dysplasia (ARVD) per modified task-force criteria, 5 (6%) had LV intramyocardial fibrosis, 7 (9%) had severe cardiomyopathy (LVEF of <40%), and 5 (6%) had abundant RV fat. No potential cause for SCA was identified in 35 (46%) patients.

## Conclusions

CMR can identify otherwise undetected possible etiologies of SCA in more than half of resuscitated patients.

## Funding

No Disclosure

